# Development and validation of a network calculator model for safety and efficacy after pancreaticoduodenectomy in the elderly patients with pancreatic head cancer

**DOI:** 10.1002/cam4.6613

**Published:** 2023-10-03

**Authors:** Ming Cai, Tong Guo, Zixiang Chen, Wuhan Li, Tian Pu, Zhiwei Zhang, Xiaorui Huang, Xinyi Guo, Yahong Yu

**Affiliations:** ^1^ Department of Biliopancreatic Surgery Tongji Hospital, Tongji Medical College, Huazhong University of Science and Technology Wuhan China; ^2^ Department of Hepatopancreatobiliary Surgery the First Affiliated Hospital of Anhui Medical University Hefei China; ^3^ Department of General Surgery, the First Affiliated Hospital University of Science and Technology of China Hefei China

**Keywords:** calculator, elderly, pancreatic head cancer, pancreaticoduodenectomy, prediction, survival

## Abstract

**Background:**

Benefiting from increased life expectancy and improved perioperative management, more elderly patients with pancreatic head cancer (PHC) underwent pancreaticoduodenectomy (PD). However, individualized predictive models for the safety and efficacy of PD is still lacking. this study aimed to developed three safety‐ and efficacy‐related risk calculators for elderly (> = 65 years) PHC patients.

**Methods:**

This study was designed with two research cohorts, namely, the training cohort and the validation cohort, and comprises four general steps: (1) Risk factors were analyzed for the incidence of postoperative complications, cancer‐specific survival (CSS), and overall survival (OS) in the training cohort (*N* = 271) using logistic and Cox‐regression analysis. (2) Nomograms were then plotted based on the above results. (3) The accuracy of the developed nomogram models was then verified with the validation cohort (*N* = 134) data using consistency index (C‐index) and calibration curves. (4) We then evaluated the efficacy of these nomograms using decision curve analysis (DCA) in both the training and validation cohorts, and ultimately constructed three online calculators based on these nomograms.

**Results:**

We identified ASA, diabetes, smoking, and lymph node invasion as predisposing risk factors for postoperative complications, and the predictive factors that affected both OS and CSS were ASA, diabetes, BMI, CA19‐9 level, and tumor diameter. By integrating the above risk factors, we constructed three nomograms on postoperative complication, CSS, and OS. The C‐index for complication, CSS, and OS were 0.824, 0.784, and 0.801 in the training cohort and 0.746, 0.718, and 0.708 in the validation cohort. Moreover, the validation curves and DCA demonstrated good calibration and robust compliance in both training and validation cohorts. We then developed three web calculators (https://caiming.shinyapps.io/CMCD/, https://caiming.shinyapps.io/CMCSS/, and https://caiming.shinyapps.io/CMOS/) to facilitate the use of the nomograms.

**Conclusions:**

The calculators demonstrated promising performance as an tool for predicting the safety and efficacy of PD in elderly PHC patients.

## 1 INTRODUCTION

Pancreatic cancer is a well‐recognized fatal digestive malignancy worldwide, which places a severe burden on global healthcare partly on account of its rising incidence every year. According to the global cancer burden data published by the International Agency for Research on Cancer (IARC) in 2020, there were approximately 495,773 new pancreatic cancer cases and 466,003 deaths caused by pancreatic cancer worldwide, making pancreatic cancer the 12th most common cancer in the world but the seventh leading cause of cancer death.^1^ Meanwhile, in China, according to the 2016 cancer incidence report published by the National Cancer Center, the incidence rate and mortality rate of PC were 7.26/105 and 6.35/105, which are consistent with the global trend.^2^

Counter to the increasing pancreatic cancer prevalence is its low survival rate, especially for pancreatic head cancer (PHC) patients. Although the postoperative survival rate of PHC patients has increased owing to improved surgical approaches and refined postoperative care management, the overall survival is not quite encouraging, with the 5‐year survival rate of PHC patients falling between 3% and 15%.^3^, ^4^ In general, the timing of therapeutic intervention is the key determinant of prognostic survival. However, as PHC is highly insidious, with a low early detection rate alongside rapid progression, most patients have already missed the best opportunity for surgery at the time of diagnosis. The survival rate of these patients is thus reduced. In addition, the radical pancreaticoduodenectomy (PD) required for PHC patients who have the chance of surgery involves the removal of multiple organs, including the stomach, pancreas, and duodenum, together with the reconstruction of the digestive tract. This surgical procedure is physically traumatic for the patient and may be followed by various postoperative complications such as pancreatic fistula, biliary fistula, postoperative bleeding, infections, and even multiorgan failure. The complication rate after PD can be up to 70%, with the incidence increasing with the patient's age.^5^ Despite the many restrictions and the availability of radiotherapy, targeted therapies, and immunotherapy as alternative treatment options, PD remains the main approach to improve long‐term patient survival.^6^ Therefore, individualized treatment protocol and surgical modality tailored based on each patient's risk profile are of utmost importance for improving clinical outcomes.

Clinicians often take the tumor‐node‐metastasis (TNM) staging system developed by the American Joint Committee on Cancer (AJCC) as a reference point when formulating treatment plans. However, this staging system only takes into account pathological‐anatomical indicators such as tumor size, tumor invasion, and lymph node metastasis, ignoring clinical features of the patient and perioperative factors, thus presenting limitations in the context of precision medicine and multiple pathogenic pathways. By contrast, the nomogram is a simple, flexible, and easy‐to‐understand multivariate prediction model that identifies key variables affecting disease prognosis based on population‐based priori risks and computes individual disease risks and survival probabilities for patients in clinical practice.^7^ In this context, the nomogram model came to our attention as a tool that has been increasingly used in the prognostic assessment of patients with various malignancies.^4^, ^5^, ^6^, ^8^, ^9^, ^10^, ^11^

The proportion of elderly patients (usually defined as those aged 65 years or older) in the global population is progressively increasing. However, there are few predictive models for the incidence of complications and survival after PD in elderly (> = 65 years) PHC patients. From the perspective of the patient's physical attributes, elderly patients typically exhibit considerably reduced functional and nutritional capacity, more frequent cognitive and mental health problems, and significantly poorer tolerance of cancer treatments. These aspects translate into longer recovery periods, increased comorbidity, and higher expected mortality in elderly PHC patients after highly invasive procedures such as PD.^12^ Therefore, when it comes to the clinical practice involving treatment decisions, the effectiveness and safety of the surgery should be carefully evaluated, taking into account the unique characteristics of the elderly patients as a vulnerable population. In addition, current models suffer from significant variation in predictive power and inconsistent inclusion factors. For example, apart from the pathological‐anatomical characteristics of the tumor, ASA, body mass index (BMI), diabetes mellitus, and smoking are also associated with poor postoperative outcomes. However, these factors have been rarely included in models by previous studies.^13^, ^14^, ^15^ Therefore, a preoperative decision‐making tool that can effectively and accurately predict the incidence of postoperative complications and survival for PHC patients that take into account the vulnerability and individual variability of the elderly patient population is greatly required by clinicians for developing optimal therapeutic plans.

In this context, this study established training and validation cohorts with patient data from three tertiary hospitals in China to screen preoperative risk factors affecting the postoperative outcomes (complications and survival) of pancreatic cancer patients. The objective is to accurately assess the safety and efficacy of PD treatment in elderly PHC patients before surgery and to provide scientific evidence for clinical decision‐making.

## 2 METHODS

### 2.1 Patients and study design

This study retrospectively included data from PHC patients treated with PD at the Department of Biliary and Pancreatic Surgery, Tongji Hospital, Tongji Medical College, Huazhong University of Science and Technology from January 2010 to January 2020 as the training cohort for establishing the nomograms. Meanwhile, in order to validate the external generalizability of these nomogram models, we also retrospectively included the information of PHC patients who received PD treatment at the First Affiliated Hospital of Anhui Medical University (FAH‐AHMU) and the First Affiliated Hospital of University of Science and Technology of China (FAH‐USTC) in the same period as the validation cohort.

Curative PD is defined as the resection of all physically and microscopically visible tumors with clear margins under the microscope (r0 resection). Our study focused solely on the patients with PHC, the inclusion criteria for this study were as follows: (1) confirmed diagnosis of pancreatic ductal adenocarcinoma (PDAC) by histopathological or cytological evaluation; (2) assessed by preoperative imaging findings as eligible for PD surgery; (3) no preoperative chemotherapy or radiotherapy; and (4) no infection, hematologic disease, or inflammatory disease before surgery. And the exclusion criteria were as follows: (1) age less than 65 years; (2) previous history of malignancy in other organs; (3) lack of key study variables or follow‐up data. This study was conducted with informed consent from all participants or their legal guardians. The study protocol was reviewed and approved by the ethical committee of Tongji Hospital, Tongji Medical College, Huazhong University of Science and Technology, FAH‐AHMU, and FAH‐USTC.

### 2.2 Data collection and measurement

The standardized database in this project contains the following information modules: (1) Patients' basic information: including age, height, body weight, tobacco consumption history, history of diabetes mellitus, and family medical history; (2) Imaging findings: including tumor size, tumor density, tissue invasion, vascular invasion, and lymph node invasion profiles; (3) Surgery information: American Society of Anesthesiologists (ASA) staging, drainage approach, operation duration, intraoperative bleeding volume, intraoperative blood transfusion volume, and organ retention; (4) Postoperative monitoring: inpatient care modalities, perioperative mortality, complications, and the management thereof. The TNM staging system was referenced to the latest Standard for diagnosis and treatment of pancreatic cancer (2022 edition) issued by the National Health Commission.^16^ BMI was defined as body weight (kg) divided by height‐squared (m^2^), and cutoff points were based on the guidelines of the Working Group on Obesity in China,^17^ defined as follows (kg/m^2^): underweight (<18.5), normal weight (18.5–23.9), overweight (24–27.9), and obesity (> = 28). Postoperative hospital stay was defined as the number of days from the date of surgery to the date of discharge. Tumor size was determined by selecting the largest tumor diameter (cm) from imaging findings. CA19‐9 (μ/mL) was categorized using the tertiles of the test results of the training cohort: <=18.66, 18.66–192.22, and > 192.22.

### 2.3 Outcomes

#### 2.3.1 Postoperative complications

The postoperative complications module of the project database contains detailed records of various complications and their management. These complications include: pancreatic fistula, biliary fistula, delayed gastric emptying, postoperative bleeding, gastrointestinal fistula, liver failure, renal failure, pulmonary infection, incisional infection, urinary tract infection, cardiac arrhythmias, and heart failure. The severity of complications was assessed by combining complication handling modalities and the Clavien‐Dindo complication grading system.^18^

#### 2.3.2 Survival time

The survival time was assessed by two indicators: cancer‐specific survival (CSS) and overall survival (OS). Patients were followed up through phone calls at 3‐month intervals (every 30 days as a follow‐up month) after discharge until death or dropout (loss of follow‐up). Dropouts included changes in contact information, refusal to be interviewed, death from other diseases or accidents, and still alive at the end of this study. If the patient died, further information such as the date and cause of death was inquired; if the patient dropped out, the date of the last telephone follow‐up was used as the dropout date. CSS refers to the number of months between the date of surgery and the date of death caused by PHC (or the date of dropout). OS refers to the number of months between the date of surgery and the date of death from any cause (or date of dropout).

### 2.4 Statistical analysis

This study used Stata/SE 15.0 (StataCorp LLC., Texas, USA) and R 4.2.0 software (http://www.r‐project.org/) for data management and statistical analysis. We applied the KNN algorithm to fill in the independent variables of the training cohort. Moreover, the validation cohort was screened by propensity score matching (PSM) based on gender, ASA classification, history of diabetes, CA19‐9, maximum tumor diameter, vascular invasion, lymph node invasion, and tumor differentiation. For the comparison of baseline characteristics between the training and validation cohorts, normally distributed continuous variables were described by mean and standard deviation and were compared by independent samples *t*‐test; categorical variables were presented as number (*n*) or percentage (%) and were compared by chi‐square test. Afterward, the median survival time and 1‐, 2‐, and 3‐year survival rates of the two cohorts were described using the Kaplan–Meier method, and the differences between those two cohorts were compared using the logrank test.

Based on the data analysis results, key preoperative variables, including age, gender, ASA, diabetes, BMI, smoking history, C19‐9, tumor size, and lymph node metastasis were included as covariates. We applied binary logistic regression analysis to assess the association strength (odds ratio, OR) between key variables and postoperative morbidity (0 = no complications, 1 = with complications) and 95% confidence interval (CI). COX proportional risk model was used to assess the association between the variables of interest and survival time (OS and CSS) and to calculate Hazard ratios (HR) and 95% CI. We then constructed nomograms for different outcome variables based on the multifactorial analysis results and tested the predictive accuracy of these nomograms using C‐index and calibration curves. Differences were considered statistically significant when the two‐sided test *p* < 0.05.

## 3 RESULTS

**FIGURE 1:**
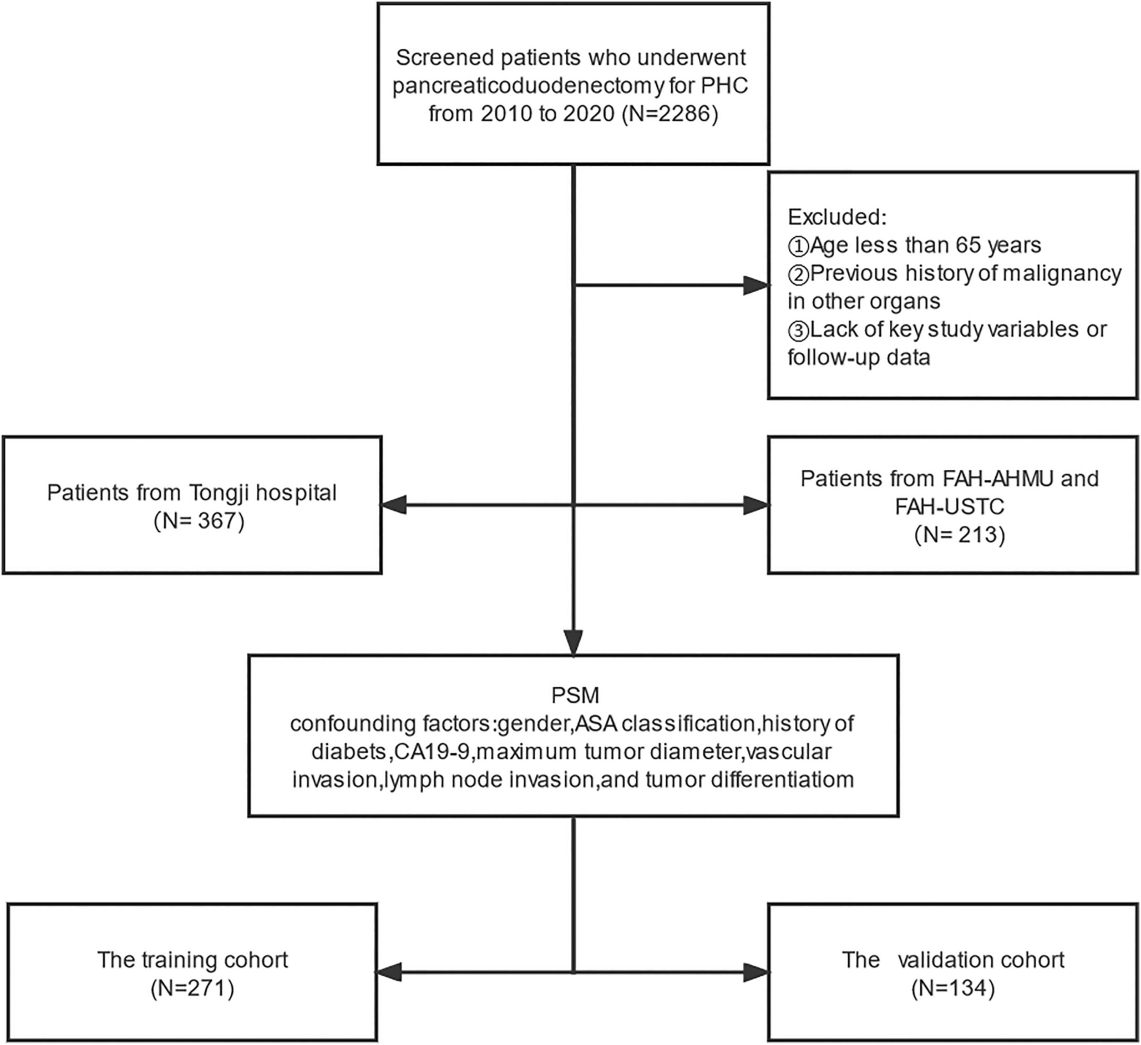
Flowchart of case selection. FAH‐AHMU, First Affiliated Hospital of Anhui Medical University; FAH‐USTC, First Affiliated Hospital of University of Science and Technology of China; ASA, American Society of Anesthesiologists; CA19‐9, carbohydrate antigen 19–9.

During the study period, a total of 2286 PHC patients received PD, including 1780 from Tongji Hospital and 506 from Anhui research sites (FAH‐AHMU and FAH‐USTC). Of these, 1706 patients were excluded due to under 65 years old, and lacked BMI and other key variables. And 271 training patients and 134 validation patients included in the were enrolled in the final analysis after initial PSM matching by gender, ASA classification, history of diabetes, CA19‐9, maximum tumor diameter, vascular invasion, lymph node invasion, and tumor differentiation. (Figure 1).

### 3.1 Demographic and clinicopathological characteristics in the training cohort

Patients in the training cohort had a mean age of 68.89 ± 3.34 years, with 155 (57.20%) males and 116 (42.80%) females. Patients in the validation cohort had a mean age of 70.39 ± 4.56 years, of which 78 (59.70%) were males, and 56 (40.30%) were females. The proportion of patients with a maximum tumor diameter < =2 cm was significantly higher in the training cohort (34.32%) than in the validation cohort (19.4%) (*p* = 0.008). Other than these factors (age and maximum tumor diameter), there were no significant differences between the two cohorts regarding demographic and clinical characteristics including ASA, history of diabetes mellitus, BMI, CA19‐9, and lymph node invasion. Among the enrolled patients, about 30% were overweight or obese, 18.82% of patients in the training cohort, and 23.88% in the validation cohort had a self‐reported history of diabetes, and nearly 85% of all PHC patients had an ASA score > = 2. Regarding surgery‐related variables, 41% of patients underwent a laparoscopic procedure, and 36% received intraoperative blood transfusions, but only about 16% had an intraoperative blood loss >600 mL (Table 1).

**TABLE 1:** Clinicopathological characteristics, operative variables, and perioperative outcomes in the training cohort and validation cohort.

Variables	Training cohort (*N* = 271)	Validation cohort (*N* = 134)	*p* Value
Clinical variables			
Age, years	68.89 ± 3.34	70.39 ± 4.56	<0.001
Sex, Male	57.20	59.70	0.631
ASA score			
1	15.13	14.93	0.119
2	58.67	49.25	
3	26.20	35.82	
Diabetes mellitus	18.82	23.88	0.235
BMI			
Underweight	13.28	7.46	0.151
Normal	56.46	55.97	
Overweight/Obesiy	30.26	36.57	
CA19‐9, μ/mL			
<=18.66	32.84	21.64	0.062
18.66–192.22	33.21	40.30	
>192.22	33.95	38.06	
Maximum tumor size, cm			
<=2.0	34.32	19.40	0.008
2–4	45.02	55.97	
>4	20.66	24.63	
Vascular invasion	7.38	9.70	0.422
Lymph node invasion	50.55	44.03	0.216
TNM stages			
IA	11.81	13.43	0.393
IB	27.68	27.61	
IIA	9.23	13.43	
IIIB	49.45	41.79	
III/IV	1.85	3.73	
Operative and pathological variables			
Tumor differentiation			
Well	8.12	7.46	0.958
Moderate	63.84	63.43	
Poor	28.04	29.10	
Operation type			
Laparoscopic	41.33	36.57	0.357
Open	58.67	63.43	
Intraoperative blood loss >600 mL	16.61	17.16	0.887
Intraoperative blood transfusion	36.16	38.06	0.709
Operation time > 180 min	88.19	85.07	0.378
Perioperative outcomes			
Postoperative stay, days	23.07 ± 12.87	22.70 ± 10.52	0.772
Postoperative morbidity			
None	63.10	56.72	0.358
Minor morbidity (Clavien‐Dindo I–II)	29.15	32.09	
Major morbidity (Clavien‐Dindo III–V)	7.75	11.19	
Postoperative 30‐day complications			
Pancreatic fistula	29.89	32.84	0.546
Delayed gastric emptying	52.03	54.48	0.642
Postoperative bleeding	8.49	11.19	0.379
Biliary fistula	1.85	2.24	0.789
Gastrointestinal fistula	0.74	1.49	0.470
Infection	5.90	5.22	0.781
Liver failure	0.37	0.00	0.481
Renal failure	0.37	0.75	0.610
Lung infection	4.43	8.21	0.122
Wound infection	0.00	0.00	
Urinary tract infection	0.00	0.00	
Arrhythmia	2.21	2.24	0.987
Myocardial ischemia/Heart failure	0.37	0.75	0.610
Perioperative mortality	3.32	0.75	0.116

Abbreviations: ASA, American Society of Anesthesiologists; BMI, body mass index; CA19‐9, carbohydrate antigen 19–9.

### 3.2 Perioperative morbidity and long‐term survival in the training cohort

Details of postoperative complications and long‐term survival rates are presented in Table 1 and Table 2. In this study, most (about 60%) patients did not develop postoperative complications, but 7.75% of patients in the training cohort and 10.33% in the validation cohort developed severe postoperative complications requiring surgical intervention or ICU management, and about 3% of these patients died in the perioperative period. Delayed gastric emptying, pancreatic fistula, and postoperative bleeding were the most common postoperative complications in PHC patients included in this study, with the same trend in the training (the incidence rates were 52.03%, 29.89%, and 8.49%, respectively) and validation (the incidence rates were 52.03%, 29.89%, and 8.49%, respectively) cohorts. As by the last follow‐up, the total mortality and cancer‐specific mortality in the training cohort were 42.73% and 37.27%, respectively, while the total mortality was higher in the validation cohort (58.21%) but cancer‐specific mortality (45.52%) was not different from that of the training cohort.

**TABLE 2:** Survival endings in the training and validation cohort.

Survival outcomes	Training cohort (*N* = 271)	Validation cohort (*N* = 134)	*p* Value
Period of follow‐up, months	24.18 ± 21.71	24.96 ± 19.90	0.738
Mortality during the follow‐up	42.73	58.21	0.005
Cancer‐specific mortality	37.27	45.52	0.125
Median CSS, month, 95% CI	44 (27, x)	30 (24, 39)	0.714
1‐year CSS rate, %	67.23	76.07	
2‐year CSS rate, %	58.97	58.00	
3‐year CSS rate, %	54.02	43.33	
Median OS, month, 95% CI	29 (17, 54)	26 (21, 33)	0.267
1‐year OS rate, %	65.28	71.57	
2‐year OS rate, %	52.56	51.39	
3‐year OS rate, %	46.15	34.13	

Abbreviations: CSS, cancer‐specific survival; OS, overall survival.

### 3.3 Multivariate analyses of predictors of postoperative complications in the training cohort

Through logistic regression analysis exploring the predictors of postoperative complications, we identified ASA, history of diabetes, smoking, and lymph node invasion as risk factors affecting the incidence of complications after PD surgery. Compared to ASA = 1, point estimates for ASA = 2 (OR = 2.51, 95% CI: 0.80–7.86) and ASA = 3 (OR = 3.58, 95% CI: 1.00–12.88) significantly increased the risk of postoperative complications with a stepwise upward trend. And patients with a history of smoking (OR = 3.20, 95% CI: 1.55–6.61) or the presence of lymph node invasion (OR = 2.11, 95% CI: 1.12–3.97) also presented an increased risk of postoperative complications (Table 3).

**TABLE 3:** Univariate and multivariate logistic regression analyses of predicting postoperative morbidity in the training cohort.

Variables	OR comparison	cOR (95% CI)	aOR (95% CI)
Age, years	Continuous	1.01 (0.94–1.09)	
Sex	Female vs. Male	0.95 (0.58–1.56)	
ASA score	2 vs. 1	4.24 (1.43–12.55)	2.51 (0.80–7.86)
	3 vs. 1	17.02 (5.44–53.26)	3.58 (1.00–12.88)
Diabetes mellitus	Yes vs. No	18.41 (7.84–43.21)	13.07 (5.17–33.08)
BMI	Underweight vs. Normal	0.89 (0.41–1.92)	
	Overweight/Obesity vs. Normal	1.20 (0.69–2.68)	
Smoking	Yes vs. No	4.15 (2.27–7.59)	3.20 (1.55–6.61)
CA19‐9, μ/mL	18.66–192.22 vs. <=18.66	1.67 (0.90–3.07)	
	>192.22 vs. <=18.66	1.22 (0.66–2.26)	
Maximum tumor size, cm	2–4 vs. <=2	1.48 (0.82–2.67)	1.01 (0.49–2.09)
	>4 vs. <=2	3.63 (1.80–7.30)	2.05 (0.87–4.85)
Lymph node invasion	Yes vs. No	2.55 (1.53–4.26)	2.11 (1.12–3.97)

Abbreviations: ASA, American Society of Anesthesiologists; BMI, body mass index; CA19‐9, carbohydrate antigen 19–9.

### 3.4 Factors associated with long‐term survival in the training cohort

Predictors affecting the two long‐term survival indicators (OS and CSS) were consistent, which were ASA, history of diabetes, BMI, CA19‐9 level, and tumor size (Table 4, Table 5). PHC patients with diabetes showed a significantly shorter postoperative CSS (HR = 3.77, 95% CI: 2.19–6.49). Higher ASA scores (HR_3 vs. 1_ = 2.99, 95% CI: 1.00–9.35) and CA19‐9 levels (HR_>192.22 vs. <=18.66_ = 1.90, 95% CI:1.02–3.56) significantly reduced the patients' postoperative CSS compared to those with lower levels. And the patients' CSS gradually declined with increasing tumor diameter (HR_2‐4 vs. <=2_ = 2.11, HR_>4 vs. <=2_ = 2.39). By contrast, in the OS‐related COX model, ASA = 3 (HR = 2.84), having diabetes (HR = 3.71), CA19‐9 > 192.22 μ/mL (HR = 1.92), maximum tumor diameter >4 cm (HR = 2.39), and overweight/obesity (HR = 3.38) were all risk factors compromising OS (*p* < 0.05).

**TABLE 4:** Univariate and multivariate Cox‐regression analyses of predicting cancer‐specific survival in the training cohort.

Variables	OR comparison	cHR (95% CI)	aHR (95% CI)
Age, years	Continuous	1.09 (1.02–1.16)	1.05 (0.99–1.12)
Sex	Female vs. Male	0.70 (0.45–1.09)	
ASA score	2 vs. 1	2.36 (0.84–6.59)	1.14 (0.39–3.33)
	3 vs. 1	9.39 (3.29–26.81)	2.99 (1.00–9.35)
Diabetes mellitus	Yes vs. No	3.84 (2.40–6.14)	3.77 (2.19–6.49)
BMI	Underweight vs. Normal	0.70 (0.29–1.67)	0.60 (0.24–1.49)
	Overweight/Obesity vs. Normal	2.91 (1.83–4.63)	2.96 (1.82–4.83)
Smoking	Yes vs. No	1.84 (1.15–2.95)	1.09 (0.64–1.84)
CA19‐9, μ/mL	18.66–192.22 vs. <=18.66	1.89 (1.02–3.48)	1.35 (0.69–2.64)
	>192.22 vs. <=18.66	2.51 (1.39–4.52)	1.90 (1.02–3.56)
Maximum tumor size, cm	2–4 vs. <=2	1.94 (1.09–3.45)	2.11 (1.13–3.93)
	>4 vs. <=2	3.20 (1.69–6.09)	2.39 (1.21–4.71)
Lymph node invasion	Yes vs. No	1.56 (1.00–2.43)	0.85 (0.50–1.45)

Abbreviations: ASA, American Society of Anesthesiologists; BMI, body mass index;CA19‐9, carbohydrate antigen 19–9.

**TABLE 5:** Univariate and multivariate Cox‐regression analyses of predicting overall survival in the training cohort.

Variables	OR comparison	cHR (95% CI)	aHR (95% CI)
Age, years	Continuous	1.07 (1.01–1.14)	1.04 (0.97–1.10)
Sex	Female vs. Male	0.77 (0.51–1.17)	
ASA score	2 vs. 1	2.32 (0.92–5.82)	1.13 (0.43–2.99)
	3 vs. 1	8.73 (3.39–22.49)	2.84 (1.01–8.00)
Diabetes mellitus	Yes vs. No	3.59 (2.30–5.62)	3.71 (2.21–6.24)
BMI	Underweight vs. Normal	0.63 (0.26–1.49)	0.53 (0.22–1.31)
	Overweight/Obesity vs. Normal	3.29 (2.13–5.07)	3.38 (2.13–5.36)
Smoking	Yes vs. No	1.62 (1.03–2.55)	0.89 (0.54–1.48)
CA19‐9, μ/mL	18.66–192.22 vs. <=18.66	1.94 (1.10–3.39)	1.42 (0.77–2.62)
	>192.22 vs. <=18.66	2.33 (1.35–4.04)	1.92 (1.07–3.44)
Maximum tumor size, cm	2–4 vs. <=2	1.96 (1.14–3.37)	1.98 (1.11–3.54)
	>4 vs. <=2	3.41 (1.87–6.22)	2.39 (1.26–4.51)
Lymph node invasion	Yes vs. No	1.65 (1.09–2.49)	0.94 (0.57–1.55)

Abbreviations: ASA, American Society of Anesthesiologists; BMI, body mass index;CA19‐9, carbohydrate antigen 19–9.

### 3.5 Construction and external validation of nomograms

**FIGURE 2:**
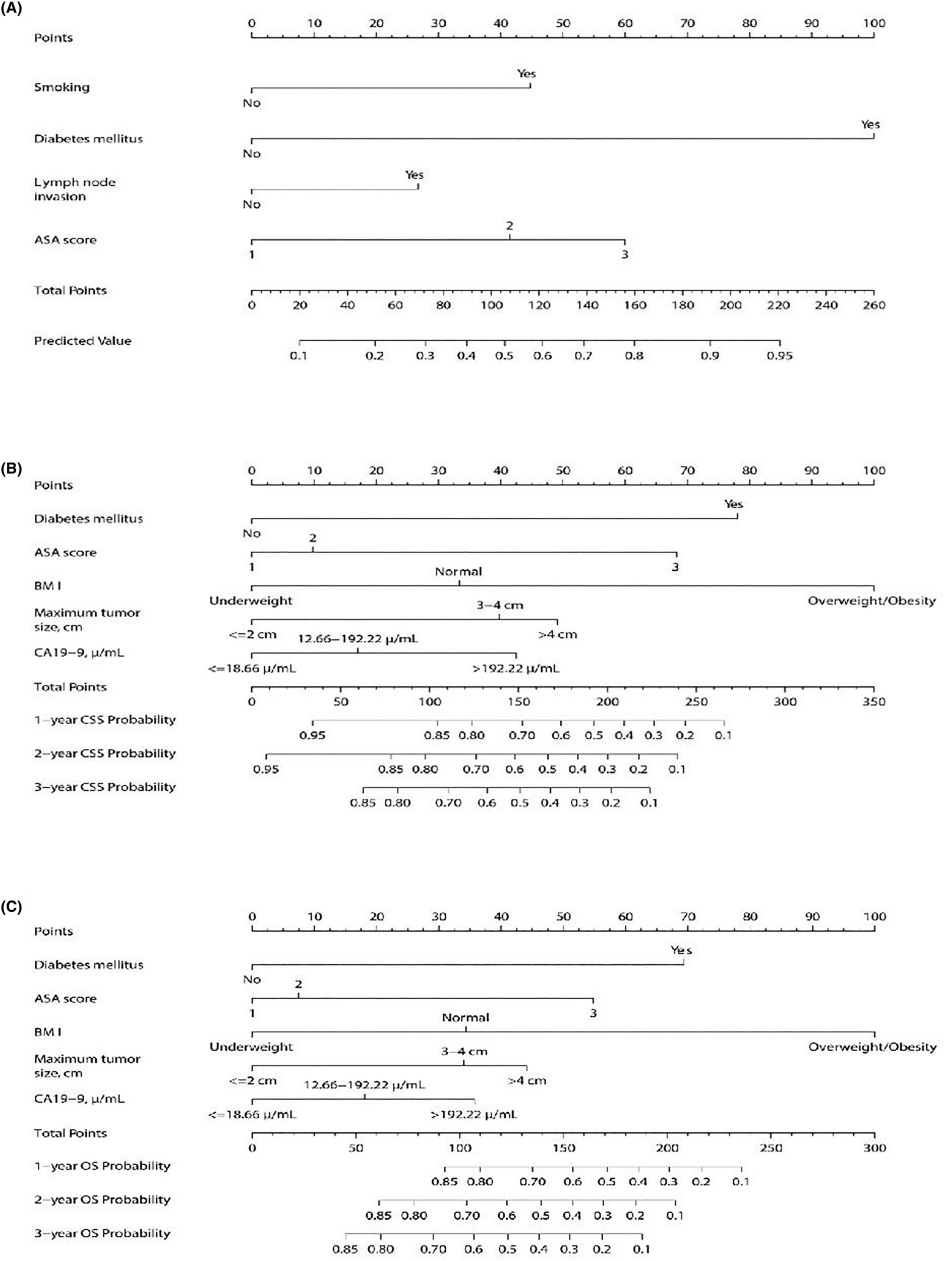
The nomogram models for the prediction of postoperative complication rates (A), CSS (B), and OS (C) for elderly patients undergoing pancreaticoduodenectomy for PHC. PHC, pancreatic head cancer; CSS, cancer‐specific survival; OS, overall survival; ASA, American society of anesthesiologists; CA19‐9, carbohydrate antigen 19–9; BMI, body mass index.

**FIGURE 3:**
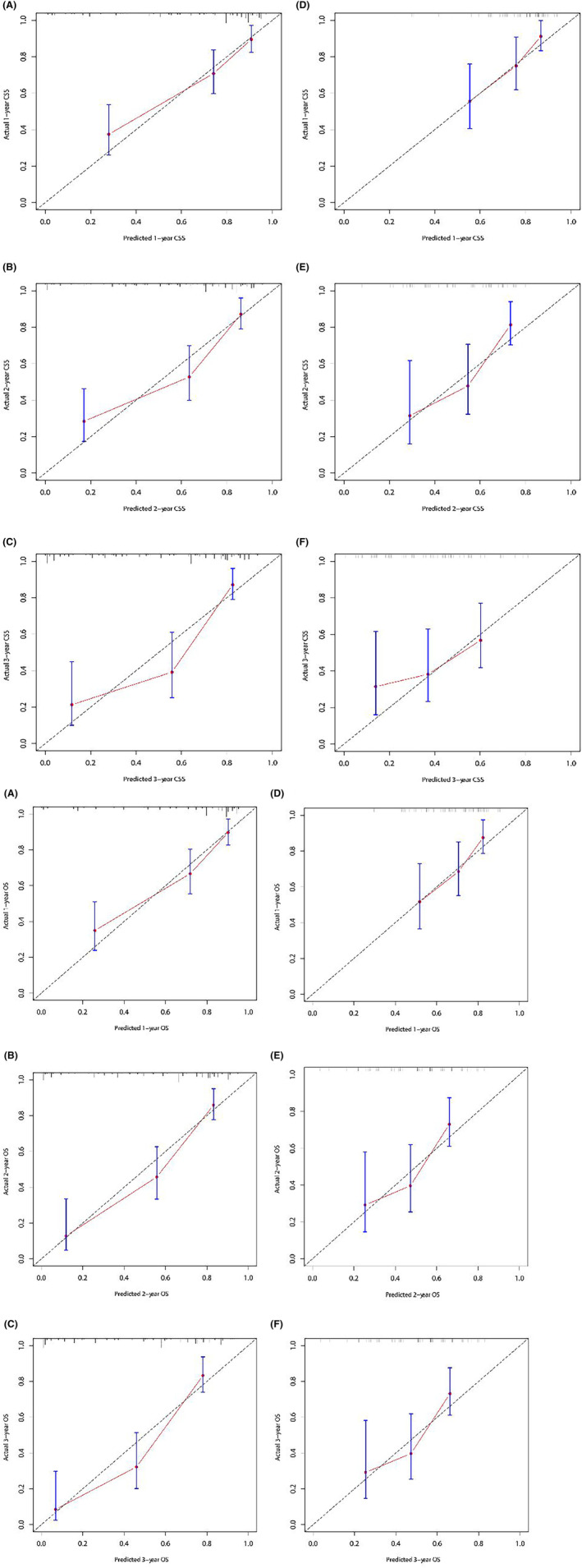
(A) Calibration plots of 1‐, 2‐, and 3‐year CSS rates for the training set (A, B, C) and validation set (D, E, F). Nomogram‐predicted probability of CSS is plotted on the x‐axis; actual CSS is plotted on the y‐axis. The gray dashed line indicates the ideal nomogram reference line. Vertical bars represent 95% confidence intervals. CSS, cancer‐specific survival. (B) Calibration plots of 1‐, 2‐, and 3‐year OS rates for the training set (A, B, C) and validation set (D, E, F). Nomogram‐predicted probability of OS is plotted on the x‐axis; actual OS is plotted on the y‐axis. The gray line indicates the ideal nomogram reference line. Vertical bars represent 95% confidence intervals. OS, overall survival.

**FIGURE 4:**
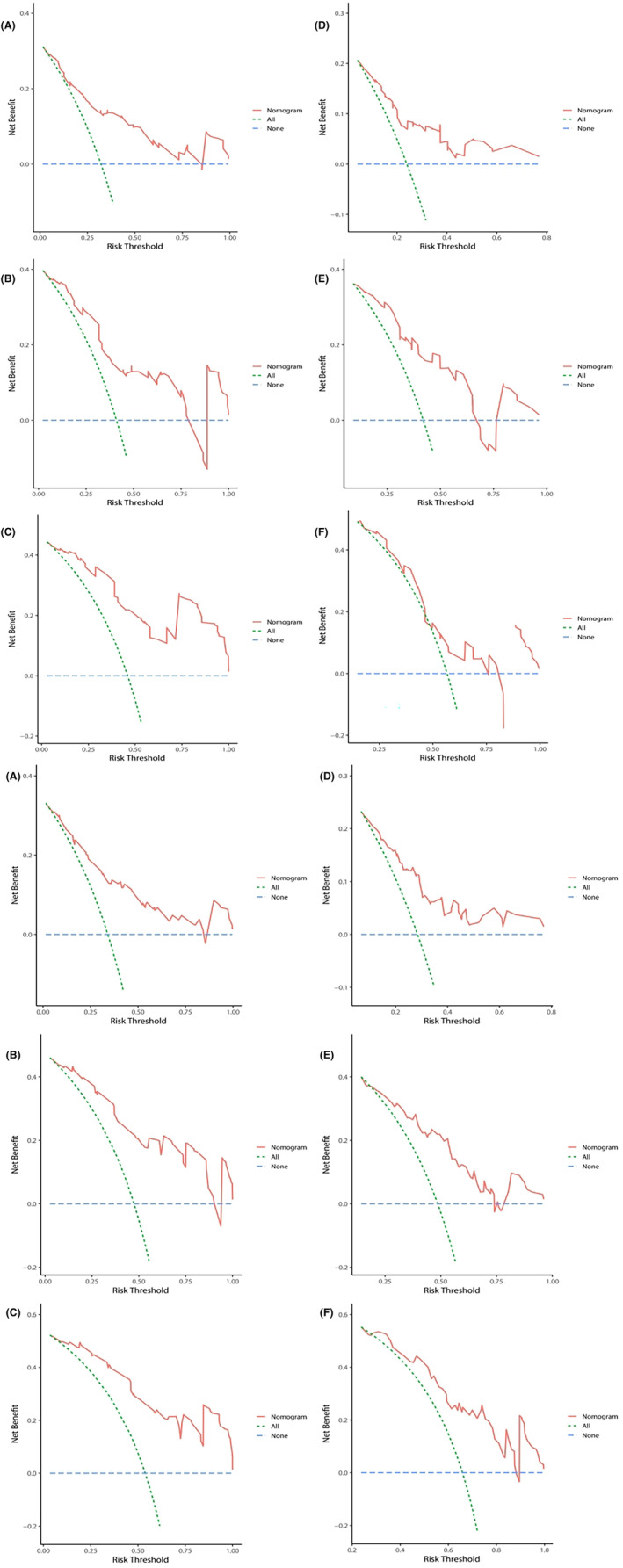
(A) Decision curve analysis (DCA) of 1‐, 2‐, and 3‐year CSS rates for the training set (A, B, C) and validation set (D, E, F). The y‐axis represents net benefit; the x‐axis shows risk threshold. The red line displays the benefit of the nomogram. CSS, cancer‐specific survival. (B) Decision curve analysis (DCA) of 1‐, 2‐, and 3‐year OS rates for the training set (A, B, C) and validation set (D, E, F). The y‐axis represents net benefit; the x‐axis shows risk threshold. The red line displays the benefit of the nomogram. OS, overall survival.

Next, we constructed nomograms (Figure 2) based on the above results and validated the prediction accuracy and internal and external compliance of these nomogram models using C‐index and calibration curves. The validation results showed that the postoperative complications, the 1‐, 2‐, and 3‐year CSS, and 1‐, 2‐, and 3‐year OS predicted by the nomograms were in good agreement with the actual outcomes, and the calibration curves fitted the reference lines, indicating high predictive validity (Figure 3a,b, Figure S1). The predictive C‐index for complication incidence, CSS, and OS in the training cohort was 0.824, 0.784, and 0.801, respectively, and the above C‐index in the validation cohort was 0.746, 0.718, and 0.708, respectively. The DCA curves also demonstrated good efficacy of the nomograms in both the training and validation cohorts (Figure 4a,b, Figure S1). Therefore, the nomograms established in this study presented good identifying capability and predictive efficacy in predicting postoperative complications and long‐term survival. Based on these three nomograms, we also built a web‐version calculator (https://caiming.shinyapps.io/CMCD/, https://caiming.shinyapps.io/CMCSS/, and https://caiming.shinyapps.io/CMOS/) to facilitate the use of these nomogram models.

## 4 DISCUSSION

The prediction of postoperative complication incidence and long‐term survival of patients is crucial for the clinical research and treatment of pancreatic cancer. In this study, we developed and validated three nomogram models for predicting post‐PD outcomes in elderly PHC patients using three‐center‐sourced clinical data by taking into account preoperative parameters, including tumor pathology indicators, demographic characteristics, and ASA score, to provide localized evidence for the employment of these risk‐predicting nomogram models. ASA score and history of diabetes were identified as corisk factors contributing to the development of complications and death after PD surgery; smoking and lymph node invasion contributed to postoperative complications without affecting long‐term survival; BMI, CA19‐9, and maximum tumor diameter were only associated with shorter CSS and OS. In addition, relevant validation has shown that these prediction models are highly accurate and are effective in guiding the development of personalized treatment plans and early interventions in clinical practice.

PD is currently considered one of the most complex and risky surgeries due to having many organs removed and many GI reconstruction anastomoses, which are associated with a high incidence of surgical complications. Postoperative complications not only share risk factors with long‐term survival but are themselves among the reference indicators for postoperative mortality, making them a key concern for clinicians.^19^ In general, the incidence of severe complications after PD surgery is <10%, which is comparable to the finding of this study. In the meantime, however, 38% of perioperative deaths after PD surgery are associated with severe complications.^20^ The incidence of delayed gastric emptying and pancreatic fistula in this study (52.03% and 29.89%, respectively) was much higher than the findings of previous studies (both close to 20%), which may be due to the differences in medical resources, treatment modalities, and postoperative nursing care levels at different study sites.^21^ Typically, delayed gastric emptying has a small impact and requires only periodic CT examinations and follow‐up assessments. By contrast, pancreatic fistulas often lead to cascading complications, such as abdominal effusion, abdominal infection, bleeding, and sepsis, and are a significant cause of postoperative mortality.^22^

In this study, we evaluated the most common postoperative complications after PD, using a nomogram to predict the likelihood of their development. The results revealed that ASA, diabetes, smoking, and lymph node metastasis were all associated with post‐PD complications. You et al. reviewed data from 1771 patients who underwent PD between 2007 and 2016, and their multifactorial analysis showed that both BMI and ASA scores were independent predictors of postoperative pancreatic fistula.^23^ The impact of diabetes on survival outcomes in pancreatic cancer patients treated with resection is still unclear, with some studies proposing no statistically significant differences in pancreatic fistula, delayed gastric emptying, infection, and perioperative death between diabetic and nondiabetic groups.^24^ However, a recent study that included 19,054 post‐PD patients found that metabolic syndrome, represented by diabetes mellitus, increased the incidence of postoperative complications (such as pulmonary embolism, acute renal failure, cardiac arrest, and delayed gastric emptying) after PD (*p* < 0.05).^25^ As another potential risk factor, smoking is not only a direct contributor to lung cancer, but also increases the incidence of postoperative anastomotic margin ulceration and even structural disruption of the anastomosis after pancreaticoduodenal surgery, which can lead to pancreatic fistula and bleeding.^26^, ^27^ Also, smoking is closely associated with infectious complications and can induce delayed gastric emptying.^28^, ^29^ And lastly, lymph node metastasis, an independent risk factor for long‐term patient survival, is associated with vascular invasion, distant metastases, and perineural invasion, worsening the nutritional status of patients while increasing the risk of postoperative bleeding.^30^

OS and CSS are indicators reflecting the long‐term survival of pancreatic cancer patients, with CSS providing a clearer picture of patient death due to this specific disease of pancreatic cancer. The independent risk factors and their risk ratios obtained through CSS are more precise and can well capture the clinical benefits of pancreatic cancer treatment. The 5‐year OS rates for pancreatic cancer patients in the United States and China are 10.8% and 7.2%, respectively,^31^ which are consistent with the commonly perceived poor prognosis of pancreatic cancer. With advances in therapeutic and paramedical approaches, the prognostic survival of pancreatic cancer patients who have undergone PD has been improved, with a 3‐year OS rate of 41.4%.^32^ For elderly patients, the 3‐year CSS rate even goes up to 53.1% for pancreatic cancer patients above > = 70 years of age.^33^ Although there were differences in the intensity of the association, the independent risk factors for OS and CSS in PHC patients were the same, namely, ASA score, history of diabetes, BMI, CA19‐9 level, and maximum tumor diameter. In this study, we found that ASA was strongly associated with long‐term survival in PHC patients who received PD. Therefore, we innovatively included it in a nomogram model for investigation. A high ASA (> = 3) often means that the patient is older, has underlying diseases, and has a higher degree of pancreatic fibrosis, resulting in a much lower survival rate in these patients.^34^ Also, previous studies have found that a higher ASA is associated with severe postoperative complications in pancreatic cancer patients, severely threatening perioperative safety and their long‐term survival.^35^

CA19‐9 level and maximum tumor diameter are closely related to the development of the tumor itself, which have been frequently investigated in previous studies.^36^, ^37^ CA 19‐9 is an important marker for pancreatic cancer. It can provide informative input for surgical decision‐making, facilitating the weighing treatment options such as surgical resection and adjuvant therapy in a high‐risk context. A lower CA 19‐9 often means a less progressive tumor and inter‐reactive prognosis for tumor progression and a higher chance the tumor is resectable, and therefore a better prognosis.^38^ Tumor size is a very important indicator in pancreatic cancer TNM staging. A larger tumor usually indicates a later diagnosis, poorer timing of therapeutic intervention, and a higher likelihood of adverse outcomes. Furthermore, a larger tumor diameter may increase intraoperative blood loss and raise the need for blood transfusion, which may lead to transfusion‐immunomodulation‐related malignancy outcomes.^39^

Previous studies have shown that BMI and diabetes are also independent risk factors for the survival of PHC patients—in addition to being a pair of related variables, each affects cancer development by unique mechanisms.^40^, ^41^ For example, high BMI increases oxidative stress, which may damage mitochondria and genetic materials; obesity is also associated with systemic inflammatory responses, promoting the secretion of pro‐inflammatory factors and chemokines and reinforcing the local invasion and cancer cell proliferation.^42^ Also, obesity alters the microenvironment of the intestinal flora, increases the metabolism and secretion of short‐chain fatty acids and endotoxins, and increases intestinal permeability, contributing to a systemic pro‐inflammatory state.^43^ Compared to nondiabetic patients, diabetic patients treated surgically for pancreatic cancer have poorer survival, which can be attributed to insulin resistance, hyperinsulinemia, and an altered cellular metabolic environment creating a favorable environment for tumor growth and metastasis. Moreover, pancreatic cancer patients with concurrent diabetes are at increased risk of tumor invasion into surrounding tissues and nerves.^24^

Nomograms are graphically illustrated statistical prediction models that predict patient‐specific risks for given outcomes. The first nomogram model was established by Memorial Sloan‐Kettering Cancer Center (MSKCC) in 2004. Compared to the traditional TNM staging system, the nomogram is more accurate in predicting prognosis and survival.^44^ In this study, we established nomograms using data from elderly PHC patients to assess the safety of PD based on the incidence of postoperative complications and the effectiveness of PD based on long‐term survival (CSS and OS). We included ASA, BMI, diabetes, smoking history, CA19‐9, tumor size, and lymph node invasion, which are more applicable to clinical decision‐making targeting this vulnerable group of elderly patients. Bootstrap internal and external validation results indicate a good agreement between the predicted values by these models and the actual observed values with respect to postoperative complications and 1‐year, 2‐year, and 3‐year OS/CSS. Our calculators, which were developed based on nomogram prediction models, feature real‐time calculations that allow clinicians to quickly and easily obtain information that predicts postoperative complication incidence and survival for patients with different risks, thereby providing clinicians with valid information to formulate different treatment plans.

To our knowledge, the present study is the first nomogram study targeting elderly PHC patients based on a multicenter design collecting tumor markers, surgical information, pathological data, and demographic data to accurately predict post‐PD complications, CSS, and OS in this patient population. Despite the strengths of this study, it is important to consider some notable limitations when interpreting the results. One key limitation is that current R software modules do not support calibration curve validation of ordered logistic models, which prevented us from exploring Clavien‐Dindo classification and outcome metrics in greater detail. Additionally, in order to emphasize early intervention and treatment planning, factors such as nursing care, postoperative indicators, and postdischarge treatment plans were not included in the follow‐up nomogram construction. However, despite these limitations, the study indicates a strong association between preoperative factors and outcomes, with a high predictive value. Although an initial PSM matching of baseline characteristics between the training and validation groups was performed, there were still some differences in age and maximum tumor diameter between the two groups. However, since other baseline characteristics showed no differences, this should have little impact on the validity of the results. It is also important to note that postoperative adjuvant chemotherapy was not accounted for in the analysis, which is a common treatment modality for certain cancers that could impact patient outcomes. Furthermore, advances in surgical techniques, perioperative management, and postoperative care over time could have influenced patient outcomes, but we addressed this by constructing models in the validation group based on risk predictors identified from the training group results and dividing the validation group into two subgroups according to the year of admission. The calibration curve results showed that the model predicted complications, CSS, and OS better in both subgroups (c‐indexes of 0.769, 0.764, and 0.712 for the <=2016 subgroup; and 0.871, 0.705, and 0.685 for the >2016 subgroup). Lastly, we included preoperative variables (such as comorbidities and tumor stage) as covariates in our analysis to minimize the potential impact of these factors. While sample collection was difficult, we aimed to balance the accuracy of the results and the feasibility of the study with long‐term sample collection.

## 5 CONCLUSION

In conclusion, although the odds of good outcomes after PD surgery in PHC patients have increased with advances in treatment and nursing care, accurate and valid risk prediction models for predicting the incidence of complications and survival after resection are still lacking for elderly PHC patients. In this study, we found that ASA score and diabetes are common risk factors affecting the development of postoperative complications and death, smoking and lymph node invasion promote postoperative complications without affecting long‐term survival, and BMI, CA19‐9, and maximum tumor diameter are independent risk factors for CSS and OS. The nomograms constructed based on the above factors can effectively predict postoperative complications and survival in elderly PHC patients, providing personalized evidence for clinicians to formulate treatment plans and clinical decisions.

## AUTHOR CONTRIBUTIONS

**Ming Cai:** Data curation (equal); formal analysis (equal); methodology (equal); project administration (equal); writing – original draft (equal). **Tong Guo:** Data curation (supporting); investigation (supporting); resources (supporting). **Zixiang Chen:** Data curation (supporting); investigation (supporting); resources (supporting). **Wuhan Li:** Data curation (supporting); investigation (supporting); resources (supporting). **Tian Pu:** Investigation (supporting); resources (supporting). **Zhiwei Zhang:** Software (supporting); validation (supporting). **Xiaorui Huang:** Software (supporting); validation (supporting). **Xinyi Guo:** Software (supporting); validation (supporting). **Yahong Yu:** Conceptualization (lead); funding acquisition (lead); supervision (lead); writing – review and editing (lead).

## CONFLICT OF INTEREST STATEMENT

No benefits in any form have been received or will be received from a commercial party related directly or indirectly to the subject of this article.

## ETHICS STATEMENT

The institutional review board of the Tongji Hospital, Tongji Medical College, Huazhong University of Science and Technology, the First Affiliated Hospital of Anhui Medical University (AHMU) and the First Affiliated Hospital of University of Science and Technology of China (USTC) approved this study.

## Supporting information


Figure S1

## Data Availability

The data that support the findings of this study are available.
