# Supplementary material for: Development and validation of a network calculator model for safety and efficacy after pancreaticoduodenectomy in the elderly patients with pancreatic head cancer
Source: Cancer Med. 2023 Oct 3;12(19):19673–89. doi: 10.1002/cam4.6613 (PMC10587938; doi:10.1002/cam4.6613)
Supplement: Supplementary file 1 — Figure S1 [file CAM4-12-19673-s001.docx]

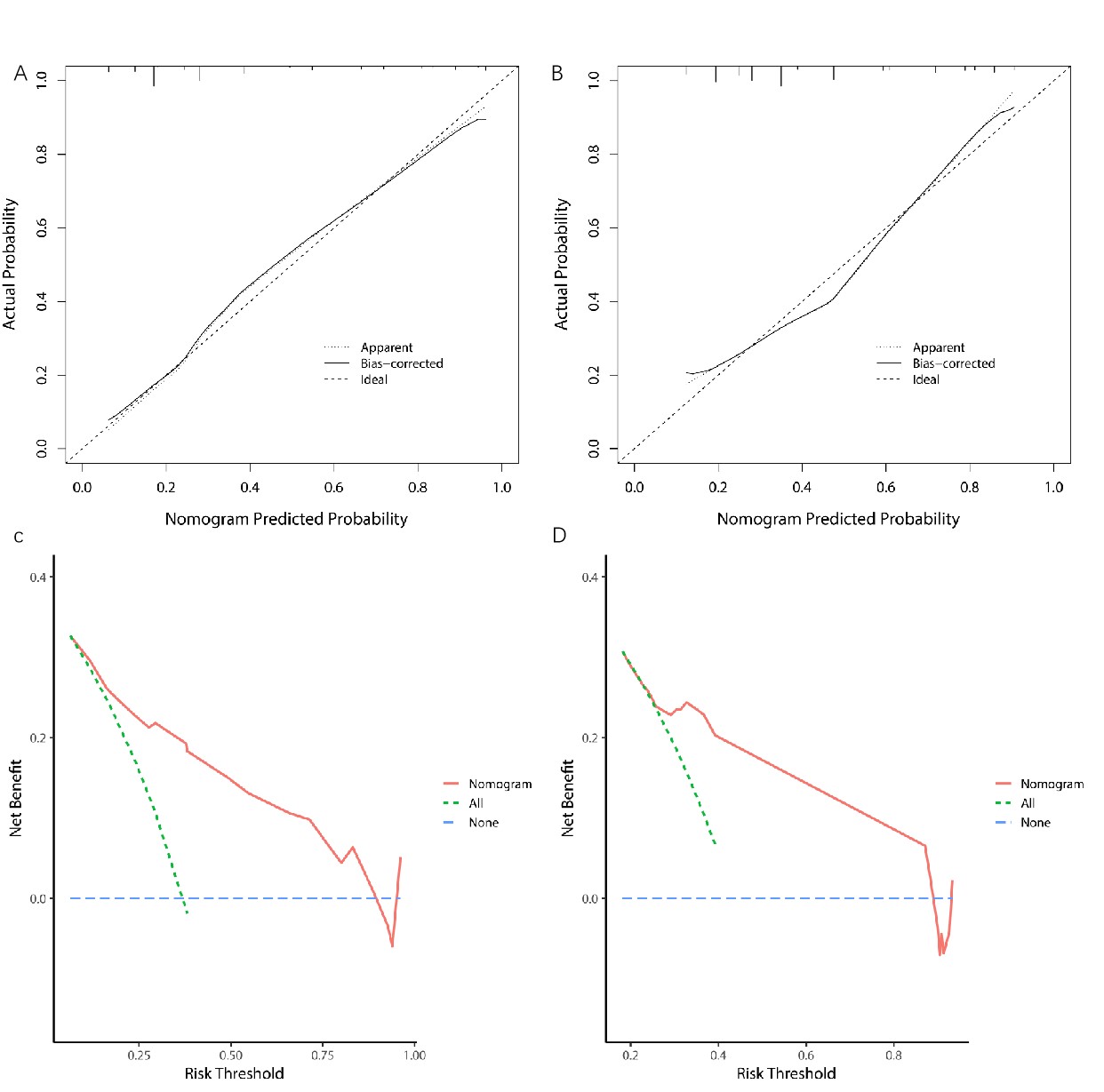


**Supplementary Figure 1**. Calibration plots of postoperative complication rates for the training set (A) and validation set (B). Nomogram-predicted probability of major morbidity is plotted on the X-axis; actual probability is plotted on the Y-axis. The dotted line (Apparent) indicates the ideal nomogram reference line. Decision curve analysis (DCA) of postoperative complication rates for the training set (C) and validation set (D). The y-axis represents net benefit, the x-axis shows risk threshold. The red line displays the benefit of the nomogram.
